# Overcoming Opacity: The Role of Intraoperative OCT in Complex Corneal and Anterior Segment Surgery

**DOI:** 10.3390/bioengineering13010015

**Published:** 2025-12-25

**Authors:** Natalie di Geronimo, Antonio Moramarco, Vito Romano, Maurizio Mete, Luigi Fontana

**Affiliations:** 1 IRCCS Azienda Ospedaliero-Universitaria di Bologna, 40138 Bologna, Italy; natalie.digeronimo@icloud.com (N.d.G.); mau.mete@gmail.com (M.M.); luifonta@gmail.com (L.F.); 2 Ophthalmology Unit, Dipartimento di Scienze Mediche e Chirurgiche, Alma Mater Studiorum University of Bologna, 40126 Bologna, Italy; 3 Eye Unit, Department of Medical and Surgical Specialties, Radiological Sciences and Public Health, University of Brescia, 25121 Brescia, Italy; vito.romano@gmail.com; 4 Eye Unit, ASST Spedali Civili di Brescia, Piazzale Spedali Civili, 25123 Brescia, Italy

**Keywords:** intraoperative OCT, anterior segment surgery, corneal surgery, opaque cornea

## Abstract

Intraoperative optical coherence tomography (iOCT) has emerged as a pivotal technology in anterior segment surgery, particularly in cases limited by corneal opacity, edema, or altered anatomy. By providing real-time, cross-sectional imaging, iOCT enables surgeons to visualize otherwise hidden structures and to perform critical intraocular maneuvers with greater precision and safety. Its integration into the surgical microscope allows continuous monitoring of tissue–instrument interaction, transforming traditionally “blind” procedures into image-guided interventions. This review highlights the role of iOCT in endothelial keratoplasty, deep anterior lamellar keratoplasty (DALK), management of acute corneal hydrops, synechiolysis, glaucoma drainage device implantation, and ocular trauma. In endothelial procedures, iOCT helps confirm Descemet membrane removal, graft orientation, and resolution of interface fluid. In DALK, it facilitates accurate cannula placement, stromal depth assessment, and evaluation of leucoma extension to guide surgical strategy. During hydrops management, iOCT supports precise air/gas injection and compression suture placement. Additionally, it enhances safety in synechiolysis, shunt implantation, and repair of traumatic corneal injuries.

## 1. Introduction

Anterior segment optical coherence tomography (AS-OCT) was first described in 1994 by Izatt et al. [1] as a key imaging tool for the diagnosis and follow-up of corneal and anterior segment disorders. Thanks to its high resolution, AS-OCT provides near-histologic cross-sectional images that support clinical evaluation and influence both medical and surgical decision-making. Over the past decade, OCT technology has evolved remarkably, transforming from a purely diagnostic tool into a true intraoperative guidance system that enhances surgical precision. The introduction of intraoperative OCT (iOCT) into the surgical field was a natural evolution, offering real-time feedback on tissue anatomy and direct guidance of surgical maneuvers [2]. Microscope-mounted configurations improved stability and probe control, while the advent of microscope-integrated OCT (MIOCT) represented a major advance, enabling continuous real-time visualization of tissue–instrument interaction through a heads-up display [3,4]. Initially used in vitreoretinal surgery [5,6,7,8,9], iOCT is now widely applied in glaucoma [10], cataract [11], and corneal transplantation procedures [12]. In corneal surgery, it provides enhanced visualization of stromal planes, guides dissection depth, and confirms graft adhesion during lamellar and endothelial keratoplasty, thus improving safety and precision in anterior segment surgery [2]. Despite its growing use, the full potential of iOCT in anterior segment surgery remains underexplored, particularly in cases where corneal opacity or anatomical alterations limit direct visualization. In such situations, the images provided by the device allow the surgeon to overcome corneal opacity and safely perform intraocular maneuvers within the anterior chamber, ensuring proper completion of the surgery. For these reasons, this manuscript is conceived as a narrative, experience-based review, aimed at addressing a relevant gap in the current literature: while previous reviews have primarily focused on the technical aspects of iOCT in specific surgical settings—most notably lamellar corneal surgery—there is still a lack of comprehensive overviews specifically dedicated to the intraoperative use of iOCT in complex anterior segment procedures performed under limited visualization, such as corneal opacity, edema, or altered anatomy. In contrast to prior works, including Moramarco et al. [2], which provided a user-oriented technical guide for lamellar keratoplasty, the present review expands the perspective across multiple anterior segment scenarios, integrating published evidence with real-world intraoperative examples to highlight how iOCT directly influences surgical decision-making, safety, and feasibility in challenging clinical conditions.

## 2. Endothelial Keratoplasty in Advanced Corneal Edema

Descemet’s stripping automated endothelial keratoplasty (DSAEK) and Descemet membrane endothelial keratoplasty (DMEK) are currently the preferred surgical techniques for the treatment of corneal endothelial disorders [13,14]. Both procedures have demonstrated excellent outcomes in terms of corneal clarity, recovery of visual acuity, and low rates of immunologic rejection. Compared with traditional penetrating keratoplasty (PK), these posterior lamellar approaches offer faster visual rehabilitation, reduced risk of rejection and postoperative astigmatism, and better structural integrity of the globe [15,16]. Given these advantages, their indications have progressively expanded to include advanced cases and eyes with severe corneal edema, where visualization of the anterior chamber is markedly reduced or even absent. In such challenging situations, intraoperative guidance—particularly through microscope-integrated OCT (iOCT)—can provide critical real-time feedback, allowing surgeons to perform the procedure safely despite limited optical transparency.

**Figure 1:**
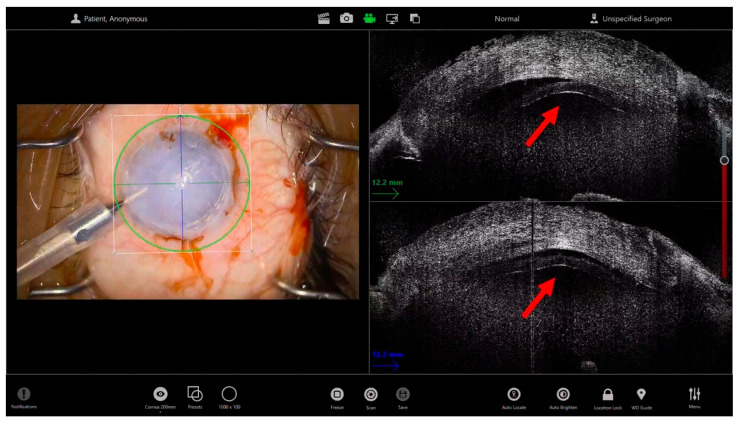
Microscope-integrated intraoperative optical coherence tomography (iOCT) guidance during endothelial keratoplasty in an eye with severely limited corneal transparency. The left panel shows the surgical microscope view, characterized by marked corneal edema and poor visualization of the anterior chamber, which prevents direct assessment of graft position and interface status. The right panels display corresponding cross-sectional iOCT images acquired intraoperatively. The red arrows highlight the interface between the donor endothelial graft and the host posterior stroma, allowing clear identification of graft position and apposition despite the opaque cornea. iOCT enables detection of residual interface fluid and confirmation of graft adherence in real time, guiding intraoperative maneuvers such as corneal massage or intraocular pressure adjustment to optimize graft attachment.

During Descemet’s Stripping Automated Endothelial Keratoplasty (DSAEK), iOCT offers valuable feedback throughout the entire procedure. The iOCT enables the surgeon to confirm the complete removal of the recipient Descemet membrane, even when this step cannot be clearly visualized under the microscope. It also helps assess the smoothness and regularity of the posterior stromal surface, ensuring optimal conditions for donor adhesion. Once the donor lenticule is introduced into the anterior chamber, iOCT can monitor its orientation and centration in real time (Figure 1). This is particularly useful when corneal opacity prevents direct visualization of the graft edge or the interface [17]. After positioning, iOCT enables the precise identification of residual interface fluid between the graft and the host stroma—one of the primary causes of postoperative detachment (REF). The surgeon can then perform targeted corneal massage or adjust intraocular pressure until complete graft apposition is confirmed. Before concluding the procedure, iOCT can verify uniform graft adherence and central thickness, providing objective confirmation of successful graft attachment even in the absence of a clear surgical view [18] (Video S1).

**Figure 2:**
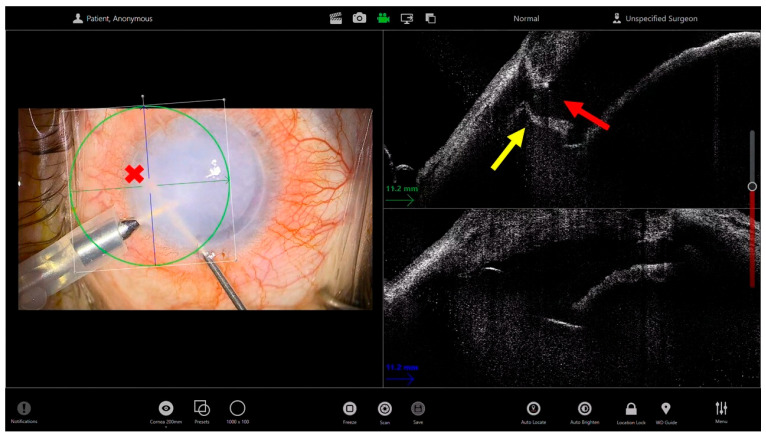
Microscope-integrated intraoperative OCT (iOCT)–guided iridectomy through an opaque cornea. The left panel shows the surgical microscope view with limited visualization of the anterior chamber due to corneal opacity; the red cross marks the intraocular position of the vitreous cutter based on the external surgical view alone. The right panels display the corresponding intraoperative OCT cross-sectional images. The yellow arrow identifies the iris plane, while the red arrow indicates the vitreous cutter tip within the anterior chamber. iOCT allows precise confirmation of the inferior positioning of the vitreous cutter and its effective contact with the iris plane, despite the lack of direct optical visualization. This real-time anatomical feedback enhances surgical safety by ensuring correct instrument depth and orientation, reducing the risk of unintended damage to adjacent structures, and confirming the adequacy of iridectomy in complex cases with compromised corneal transparency.

In Descemet Membrane Endothelial Keratoplasty (DMEK), where the donor tissue is much thinner and more delicate, iOCT becomes even more indispensable in eyes with poor visibility: iOCT helps confirm the complete and clean removal of the diseased Descemet membrane, avoiding unrecognized remnants that may hinder donor adhesion. The donor graft, being a thin, translucent membrane, often remains invisible in edematous corneas. iOCT provides continuous monitoring of the scroll’s position during surgery and its configuration, allowing the surgeon to recognize the correct orientation (endothelium-down) and to guide gentle maneuvers for unfolding avoiding overmanipulation. During and after air injection, iOCT can verify the absence of residual fluid clefts and the uniform apposition of the graft to the host stroma. This reduces the risk of postoperative partial detachment and the need for rebubbling procedures [19]. Moreover, in both techniques, intraoperative OCT can be helpful in guiding the iridectomy performed with a vitreous cutter, as it allows localization of the instrument, verification of its contact with the iris, and subsequent confirmation of the iridectomy’s patency (Figure 2).

In both techniques, iOCT serves as an “invisible assistant”, translating cross-sectional images into actionable information. It increases the surgeon’s confidence in challenging cases, allows for an earlier transition from penetrating to lamellar techniques, even in eyes with minimal transparency, and ultimately contributes to safer, more predictable surgical outcomes.

## 3. Deep Anterior Lamellar Keratoplasty for Leucomatous Corneas

Deep anterior lamellar keratoplasty (DALK) is considered the procedure of choice for corneal pathologies that primarily affect the stroma while sparing the endothelium [20]. By selectively replacing diseased stromal tissue and preserving the host Descemet membrane and endothelium, DALK restores corneal transparency and surface regularity while minimizing the risks of open-sky surgery. This lamellar approach virtually eliminates the possibility of endothelial rejection, the most common cause of graft failure after penetrating keratoplasty, and significantly reduces the risk of intraoperative complications such as expulsive hemorrhage or wound dehiscence [21,22]. Furthermore, preservation of the host endothelium enhances long-term graft survival and maintains endothelial cell density. Another relevant advantage of DALK lies in its favorable refractive outcomes [23]. The technique enables the use of large-diameter grafts, improving optical quality and reducing postoperative irregular astigmatism compared with penetrating keratoplasty [24]. These features make DALK a safe and effective option for a wide range of stromal disorders, including advanced keratoconus, stromal dystrophies, and postinfectious or posttraumatic corneal scars.

**Figure 3:**
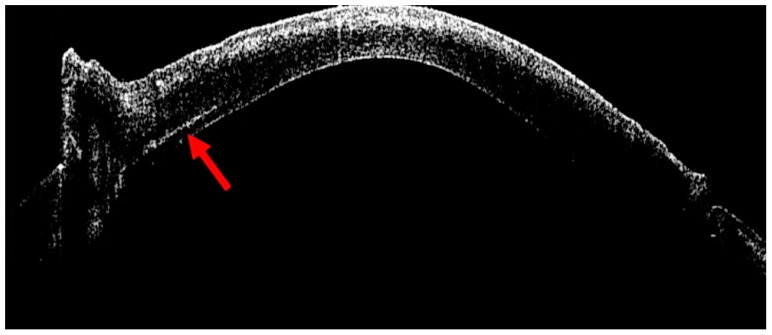
Intraoperative optical coherence tomography (iOCT) visualization of the stromal tunnel during the big-bubble technique in deep anterior lamellar keratoplasty (DALK). The cross-sectional iOCT image shows the corneal profile during cannula insertion into the deep stroma. The red arrow highlights the hyperreflective linear tract within the corneal stroma, corresponding to the stromal tunnel created by the cannula during air injection. This hyperreflective track allows precise identification of the depth and orientation of cannula placement after withdrawal, even in eyes with leucomatous or opaque corneas where conventional microscopic signs are absent. iOCT confirmation of an appropriately deep, pre-Descemetic tunnel is critical to optimize the likelihood of successful big-bubble formation while minimizing the risk of inadvertent Descemet membrane perforation.

The success of Deep Anterior Lamellar Keratoplasty (DALK) largely depends on achieving an optimal stromal dissection plane just above Descemet’s membrane, which is traditionally obtained through the “big bubble” technique [25]. This method involves inserting a cannula into the deep stroma to inject air into the corneal tissue, thereby creating a cleavage plane between the posterior stroma and the Descemet’s membrane. A key factor for successful bubble formation is the accurate placement of the cannula at the appropriate stromal depth. In eyes with good corneal transparency, correct cannula positioning can often be inferred from the appearance of radial stromal folds radiating from the cannula tip during air injection. However, in leucomatous or opaque corneas, this characteristic sign is frequently absent, making it more challenging to determine the correct insertion depth. In such situations, intraoperative OCT can be extremely helpful for verifying the cannula’s position within the stroma. After cannula removal, a hyperreflective linear track can typically be observed within the stroma, indicating the exact depth of insertion (Figure 3). Moreover, iOCT can sometimes visualize the cannula tip in real time during advancement, producing the characteristic “seagull wing” sign, which represents the hyperreflective appearance of the metallic tip as it approaches the pre-Descemetic plane [2]. This real-time feedback allows the surgeon to fine-tune the depth of dissection and optimize the chance of achieving a successful big bubble while minimizing the risk of Descemet membrane perforation.

Another application of iOCT during anterior lamellar keratoplasty is the exploration and assessment of the transparency of the deeper layers. Indeed, during the preoperative and intraoperative evaluation of corneal leucomas, assessment of the integrity of the deeper layers is often hindered by the presence of the anterior leucoma and by backscattering phenomena that may occur on anterior segment OCT. In the intraoperative setting, a superficial stromectomy can be performed, thereby improving visualization of the deeper layers and allowing a more adequate evaluation of the deep stroma. At this point, the deep stroma can also be analyzed using intraoperative OCT to confirm tissue integrity, providing the surgeon with useful information to decide whether to proceed with lamellar surgery or convert to penetrating keratoplasty. Moreover, assessment of the depth of the leucoma is instrumental in evaluating the likelihood of achieving a type 1 bubble. Indeed, deeper leucomas more frequently result in the formation of a type 2 bubble, which may significantly increase the technical difficulty of the procedure as well as the risk of unintended conversion [26]. Conversely, identification of a deep leucoma may influence the surgeon’s decision-making process, leading them to choose manual dissection rather than attempting bubble formation.

## 4. Management of Corneal Hydrops

**Figure 4:**
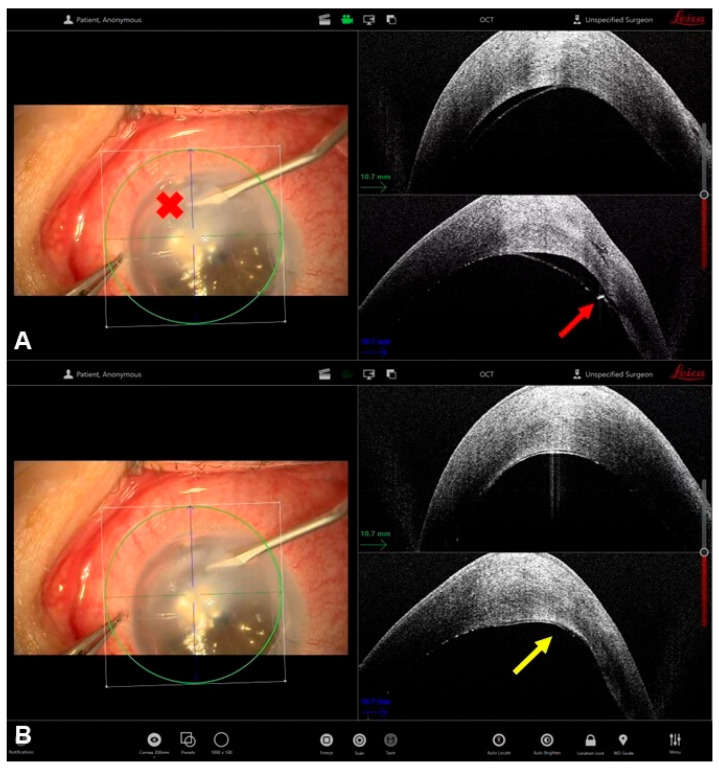
Intraoperative OCT–guided management of acute corneal hydrops with intrastromal drainage. Panels A and B show the surgical microscope view (**left**) and the corresponding intraoperative OCT cross-sectional images (**right**). In panel (**A**), the red cross on the microscope image indicates the position of the blade tip during intrastromal paracentesis, while the red arrow on the iOCT image identifies the exact correspondence of the blade within the corneal stroma, allowing precise confirmation of its depth and trajectory despite severe corneal edema and poor visualization. This real-time feedback enables safe advancement of the instrument toward the plane between the posterior stroma and Descemet membrane. In panel (**B**), the yellow arrow highlights the resolution of the Descemet membrane detachment following controlled fluid drainage, with reapposition of Descemet membrane to the posterior corneal stroma.

Acute corneal hydrops represents a complication of advanced ectatic disorders, mainly keratoconus, characterized by a sudden rupture of Descemet’s membrane and subsequent stromal imbibition of aqueous humor. This results in marked stromal edema and a profound reduction in corneal transparency, which can significantly delay visual rehabilitation and complicate future keratoplasty [27]. In recent years, intraoperative OCT has emerged as a valuable adjunct for both diagnostic assessment and surgical management of hydrops. Intracameral air or gas injection (descemetopexy) has been proposed as an effective approach to promote reapposition of the ruptured Descemet membrane and accelerate the resolution of stromal edema in acute corneal hydrops [28]. The goal of this technique is to create a temporary tamponade effect by filling the anterior chamber with air or gas (such as SF_6_ or C_3_F_8_), thereby pushing the detached Descemet membrane back toward the posterior stroma and facilitating its reattachment. Intraoperative OCT plays a pivotal role in this procedure by allowing precise identification of the Descemet break and the extent of detachment before the injection [29]. During air injection, iOCT provides real-time visualization of the anterior chamber fill, helping to prevent overinflation and minimizing the risk of pupillary block or endothelial touch. The surgeon can directly observe the contact interface between the air bubble and the detached membrane, confirming adequate tamponade and immediate reduction in stromal edema. Residual pockets of intrastromal fluid can also be identified and, if necessary, drained through a controlled paracentesis or gentle corneal massage under OCT guidance (Figure 4) [29].

In addition to air injection, iOCT has proven instrumental during compression suture placement, a technique aimed at approximating the edges of the Descemet break and promoting stromal compaction. Real-time cross-sectional imaging enables surgeons to identify the extent and depth of intrastromal fluid clefts, confirm the location of the Descemet membrane tear, and guide the optimal depth of partial-thickness sutures (typically 50–60% of stromal depth) [30]. During suture application, iOCT provides dynamic visualization of stromal compaction and collapse of intrastromal fluid pockets, allowing intraoperative adjustment of suture tension to achieve uniform compression. This real-time feedback is particularly valuable in markedly edematous or opaque corneas, where conventional slit-lamp visualization is inadequate [31]. The surgeon can observe the progressive reduction in stromal thickness and closure of intrastromal clefts directly on iOCT, ensuring adequate mechanical support without excessive tension that might induce Descemet’s membrane trauma. Postoperatively, iOCT or AS-OCT follow-up enables objective monitoring of corneal thickness stabilization and Descemet’s stromal reattachment, guiding optimal suture removal timing and preventing hydrops recurrence. In cases where both compression sutures and descemetopexy are employed, iOCT helps to optimize their sequence and synergy, ensuring that the sutures are placed at an appropriate depth and that the intracameral bubble provides uniform posterior support along the rupture line [32].

By offering continuous anatomical feedback, iOCT-guided air injection increases the precision and safety of descemetopexy, particularly in eyes with dense stromal edema or poor anterior chamber visibility. This image-guided approach not only shortens the recovery time and promotes faster resolution of corneal edema but also reduces the need for later keratoplasty, improving long-term structural and visual outcomes.

## 5. Synechiolysis

Peripheral anterior synechiae (PAS) can develop as a sequela of intraocular inflammation, trauma, or previous corneal surgery such as penetrating keratoplasty (PK). These adhesions between the peripheral iris and the corneal endothelium or graft interface may compromise the aqueous outflow, leading to secondary angle closure and progressive endothelial decompensation. Surgical synechiolysis is often required to restore anterior chamber anatomy and prevent glaucoma or graft failure; however, this procedure can be technically demanding in eyes with peripheral corneal opacification, where direct visualization of the angle is limited [33].

**Figure 5:**
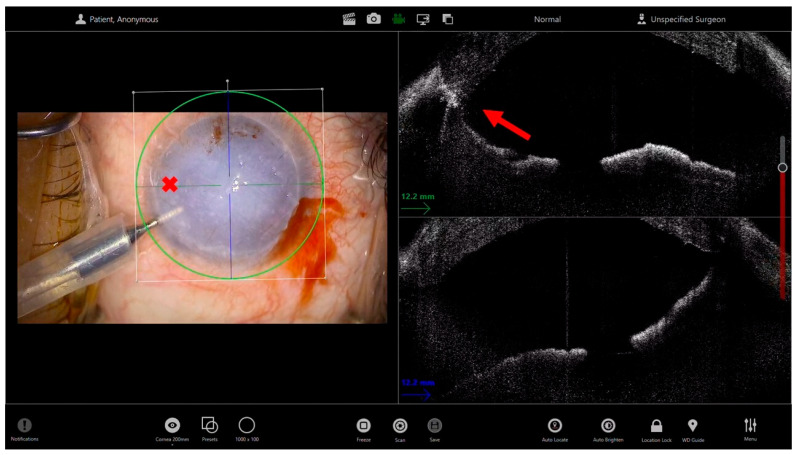
Intraoperative OCT–guided synechiolysis in the presence of peripheral corneal opacity. The left panel shows the surgical microscope view with limited visualization of the anterior chamber due to corneal opacity. The red cross identifies the corneal meridian corresponding to the location of peripheral anterior synechiae, which cannot be reliably visualized through the operative microscope alone. The right panels display the corresponding intraoperative OCT cross-sectional images acquired along this meridian. The red arrow highlights the iridocorneal adhesion, allowing precise localization of the synechia and delineation of its extent. By correlating the external surgical view with cross-sectional OCT imaging, iOCT enables accurate targeting of synechiolysis, confirms separation of the iris from the corneal endothelium or graft–host junction, and reduces the risk of unintended trauma to adjacent structures.

In such cases, iOCT serves as an invaluable tool, providing real-time, cross-sectional visualization of anterior segment anatomy. iOCT enables the precise localization and mapping of synechial adhesions that may not be visible clinically through the operative microscope, allowing surgeons to plan the extent and depth of dissection with accuracy (Figure 5). During the procedure, iOCT confirms the progressive separation of the iris from the corneal endothelium or graft-host junction, and visualizes the reformation of the anterior chamber angle as the adhesions are lysed. Furthermore, iOCT can immediately verify the completeness of synechiolysis at the end of surgery, distinguishing residual attachments from normal anatomic structures.

## 6. Glaucoma Shunt

The implantation of glaucoma drainage devices requires precise intraoperative control to ensure correct positioning of the implant and to prevent complications such as endothelial trauma, iris touch, or malposition within the anterior chamber. This is particularly challenging in eyes with corneal opacity, gerontoxon, or distorted anterior chamber anatomy, where direct visualization through the surgical microscope may be inadequate [34].

In such cases, intraoperative OCT serves as a valuable adjunct, providing real-time cross-sectional visualization of the anterior chamber and surrounding tissues during drainage device implantation. iOCT can confirm the exact entry site and trajectory of the needle tract, ensuring the device passes through the correct scleral plane into the anterior chamber without damaging the iris or the crystalline lens in phakic patients. Once the implant is positioned, iOCT imaging verifies its tip location, angulation, and proximity to the corneal endothelium, allowing adjustments before concluding the surgery. This intraoperative feedback helps prevent postoperative complications such as endothelial cell loss or malposition-related obstruction. Overall, iOCT transforms the implantation of glaucoma drainage devices from a largely experience-based procedure into a controlled, image-guided surgery, increasing accuracy, reducing intraoperative uncertainty, and enhancing the long-term safety of filtration outcomes in eyes with compromised corneal clarity [35].

## 7. Trauma

Ocular trauma involving the cornea and anterior segment often leads to marked stromal edema, epithelial defects, or blood infiltration into the anterior chamber, all of which severely impair intraoperative visualization. In such challenging scenarios, iOCT offers a decisive advantage by providing real-time cross-sectional imaging of the anterior segment, enabling surgeons to safely navigate anatomical planes that would otherwise be obscured.

In eyes with penetrating corneal trauma, visualization of wound margins, anterior chamber depth, and residual foreign material is often limited due to corneal opacity or localized edema. Under these circumstances, iOCT enables precise assessment of wound depth and extension, and guides the placement of sutures to ensure proper wound apposition and anterior chamber reformation. Bondalapati et al. demonstrated that iOCT can accurately identify and guide the removal of intrastromal or anterior chamber foreign bodies even when the cornea is opaque, thereby minimizing further endothelial or stromal trauma [36]. Similarly, in post-keratoplasty wound dehiscence secondary to blunt trauma, iOCT provides continuous feedback on the integrity of the graft–host junction and on the configuration of the Descemet membrane. During surgical repair, it allows real-time monitoring of graft alignment, suture depth, and intracameral air tamponade, which are otherwise difficult to judge when the cornea becomes edematous or blood-stained. As reported by Chaniyara et al., this approach facilitates accurate reapposition of the corneal layers and confirms intraoperative restoration of anterior chamber depth, even in cases with complete loss of optical clarity [37].

The ability of iOCT to penetrate edematous or leucomatous corneal tissue makes it an invaluable tool in traumatic eyes with poor visibility, transforming potentially high-risk “blind” procedures into image-guided, controlled interventions. By enabling the surgeon to visualize hidden structures—such as the Descemet membrane, graft interface, or anterior chamber boundaries—iOCT significantly enhances surgical safety, reduces iatrogenic injury, and improves the likelihood of anatomical and functional recovery in complex traumatic cases.

## 8. Future Directions

Future developments in intraoperative OCT are expected to further enhance its role as a decision-support tool in anterior segment surgery. Advances in real-time image processing may enable automated tissue segmentation and quantitative tracking of corneal and anterior chamber structures, providing continuous feedback on tissue thickness and instrument proximity. The integration of artificial intelligence algorithms could facilitate intraoperative recognition of surgical planes, interface fluid, or unsafe maneuvers, supporting surgeon decision-making in complex cases. Additionally, improved acquisition speed, wider fields of view, and better management of instrument-related shadowing may further optimize intraoperative visualization. Together, these innovations may contribute to safer, more standardized, and more reproducible image-guided anterior segment surgery [38].

## 9. Conclusions

Intraoperative optical coherence tomography (iOCT) has emerged as a transformative adjunct in anterior segment surgery performed under limited visualization. By providing real-time, cross-sectional imaging of ocular structures, iOCT enables surgeons to overcome challenges imposed by corneal opacity, edema, or distorted anterior chamber anatomy, allowing image-guided precision in procedures that were previously performed with limited or indirect visual feedback. Its integration into the surgical microscope has expanded the feasibility of lamellar keratoplasty, facilitated the safe execution of complex maneuvers such as synechiolysis and glaucoma drainage device implantation, and improved intraoperative control in traumatic or severely edematous eyes (Table 1).

It should be acknowledged that the evidence supporting many current applications of iOCT in anterior segment surgery is predominantly derived from observational studies, small case series, and expert experience, with limited availability of high-level comparative data. In addition, access to microscope-integrated iOCT systems remains variable across institutions, and the technology entails costs, workflow considerations, and a learning curve that may restrict widespread adoption. Despite these limitations, across published reports and real-world clinical practice, iOCT consistently provides unique intraoperative anatomical information that is otherwise unavailable in eyes with compromised visualization, offering a level of real-time guidance that cannot be fully replicated by alternative imaging modalities such as ultrasound biomicroscopy or endoscopy.

Within this context, the contribution of the present narrative review lies not in quantitative synthesis, but in the integration of available evidence with real-life intraoperative scenarios, illustrating how iOCT directly influences surgical decision-making, enhances safety, and expands the range of anterior segment procedures that can be performed in challenging clinical conditions. As image resolution and acquisition speed continue to improve, future developments may include automated depth tracking, artificial intelligence–assisted intraoperative feedback, and integration with digital and robotic surgical platforms. These advances may further strengthen the role of iOCT as a decision-support tool in complex anterior segment surgery, bridging the gap between imaging and intervention.

## Figures and Tables

**Table 1:** Summary of clinical applications and evidence supporting intraoperative OCT in complex anterior segment surgery.

	Study Type	Sample Size	Procedure	iOCT Role
Pasricha et al. [17]	Case series	2 patients	DSAEK	Graft insertion, unfolding, tamponade, and attachment verification
Sharma et al. [19]	Prospective interventional case series	25 eyes	DMEK	Visualizing areas of PAS, retained DM tags, roll configuration and orientation, interface fluid and peripheral folds
Moramarco et al. [29]	Case series	6 eyes	Corneal hydrops drainage	Controlled drainage of the stromal dome with a 23-gauge sclerotome, with dynamic monitoring of fluid outflow
Siebelmann et al. [30]	Case series	2 patients	Corneal hydrops drainage	iOCT-guided puncture and drainage of intrastromal fluid pockets combined with anterior chamber sulfur hexafluoride-fill and pre-descemetic sutures
Kaur et al. [31]	Prospective interventional case series	7 patients	Corneal hydrops drainage	Assess the morphological features of the hydrops, titrate the magnitude of suture tightness and confirm the depth of suture placement
Petrovic et al. [33]	Case report	1 patient	Synechiolysis	Detect non-clinically visible synechiae and confirm complete synechiolysis
Platas Moreno et al. [35]	Case report	1 patient	Ex-PRESS^®^ implant	Locate the correct placement of implants
Bondalapati et al. [36]	Case report	1 patient	Intraocular foreign body removal	Localization of the foreign body and confirmation that there was no remaining foreign body
Chaniyara et al. [37]	Case report	1 patient	Traumatic wound dehiscence repair in DALK	Visualization of donor tissue separated from host Descemet’s membrane, peripheral iridocorneal touch and distorted graft–host junction

## Data Availability

The original contributions presented in the study are included in the article/Supplementary Material, further inquiries can be directed to the corresponding author.

## Supplementary Materials

The following supporting information can be downloaded at: https://www.mdpi.com/article/10.3390/bioengineering13010015/s1, Video S1: DSAEK OCT guided.

## Abbreviations

The following abbreviations are used in this manuscript:

MIOCT	Microscopy integrated optical coherence tomography
iOCT	Intraoperative OCT
PAS	Peripheral anterior synechiae
DALK	Deep anterior lamellar keratoplasty
